# Care reality of menopausal women in Germany: healthcare research using quantitative (SHI claims data) and qualitative (survey) data collection

**DOI:** 10.1007/s00404-022-06457-9

**Published:** 2022-03-07

**Authors:** Petra Stute, Helena Eversheim, Diethe Ortius-Lechner, Melanie May, Chiara Feig

**Affiliations:** 1 Gynaecological Endocrinology and Reproductive Medicine, University Women’s Hospital Inselspital, Friedbuehlstrasse 19, 3010 Bern, Switzerland; 2 Besins Healthcare Germany GmbH, Ullsteinhaus, Mariendorfer Damm 3, 12099 Berlin, Germany; 3 HGC Healthcare Consultants GmbH, Graf-Adolf-Platz 15, 40213 Düsseldorf, Germany

**Keywords:** Menopause, Hormone replacement therapy (HRT), Forsa survey, Statutory Health Insurance (SHI) claims data, Real-world evidence (RWE)

## Abstract

**Purpose:**

The transition from the fertile phase of life to menopause is associated with numerous physical changes. Hormone replacement therapy (HRT), as the most effective and efficient form of drug treatment, involves the use of oestrogens and progestins with the aim of increasing health-related quality of life through symptom reduction, sleep improvement and affect enhancement.

**Methods:**

The medical care situation and disease burden of menopausal women was investigated by means of a survey of 1000 women aged 45–60 years on the topics of quality of life, menopause and HRT and a quantitative, longitudinal healthcare study based on an anonymised and age- and sex-adjusted Statutory Health Insurance (SHI) routine data set with approximately four million anonymous insured persons per year.

**Results:**

Out of more than half a million women aged 35–70 years, and with statutory health insurance, (*n* = 613,104), 14% (*n* = 82,785) had climacteric disorder documented as a first diagnosis in 2014. The proportion of women with the climacteric disorder, who were prescribed HRT on an outpatient basis, was 21%; according to the forsa survey, 50% of the women surveyed felt moderate to poorly/very poorly informed about treatment options.

**Conclusion:**

Findings from the health insurance research conducted with different data sources (survey and SHI claims data) indicate the need for increasing awareness and providing an early and informative education on HRT and its risks and benefits.

## Introduction

Menopause is the last, spontaneous menstrual period in a woman's life and occurs at an average age of 51 years [1]. Menopause is associated with a decrease in ovarian function, as reflected in the reduced sex steroid biosynthesis. The multi-year transition from the fertile phase of life to menopause is associated with numerous physical changes [2]. Most symptoms, which occur with varying intensity, are transient in nature [3]. However, the cessation of ovarian function and resulting hormone deficiency, if left untreated, may result in long-term consequences of the disease, such as osteoporosis [4], coronary heart disease [5] and cognitive impairment [6]. The most important acute accompanying symptoms of menopause, some of which severely impair quality of life, include hot flashes and sweating, sleep disturbances, mood swings, depression, fatigue, memory impairment, sexual dysfunction, urogenital complaints, muscle and joint discomfort, weight gain and skin and hair changes [3, 7, 8]. Conventional hormone replacement therapy (HRT), complementary and alternative medicine (CAM) and nonhormonal pharmacotherapy are available for the treatment of the menopausal syndrome. HRT is the most effective treatment modality [9–12] for reducing symptoms such as hot flashes, improving sleep quality and decreasing the severity of depression [13, 14].

As a result of the Women's Health Initiative (WHI) study [15, 16], the largest prospective randomised placebo-controlled study on HRT, there was a dramatic collapse in the number of HRT prescriptions [17], despite the study’s subsequent more precise conclusions [18]. Even though upon closer examination, the supposedly high health risks (cardiovascular disease, breast cancer) conveyed in these studies turned out to be much smaller than perceived by the public [12, 18], the proportion of HRT prescribed decreased to less than 10% and uncertainty among physicians and patients increased [19–22]. However, not prescribing HRT may lead to a higher burden of disease when symptoms remain untreated, thereby reducing the quality of life. Findings stress that this can lead to higher healthcare costs and increased incapacity for work [17, 23–26].

The aim of this healthcare research was to map the medical care situation of menopausal women in Germany and, consequently, investigate the burden of disease. Primary data (survey) and secondary data (Statutory Health Insurance (SHI) claims data) were used for this purpose.

## Methods

### Study design and sample

#### Menopausal women survey

The results of a survey of 1000 women aged 45–60 years on the topics of quality of life, menopause and HRT were used to assess the reality of care from the patients’ perspective. The survey was conducted from 6 to 12 May 2020, using the online survey panel forsa.omninet. The primary data obtained are representative and can be applied to the overall population of women aged 45–60 in Germany.

#### SHI claims data

**Fig. 1:**
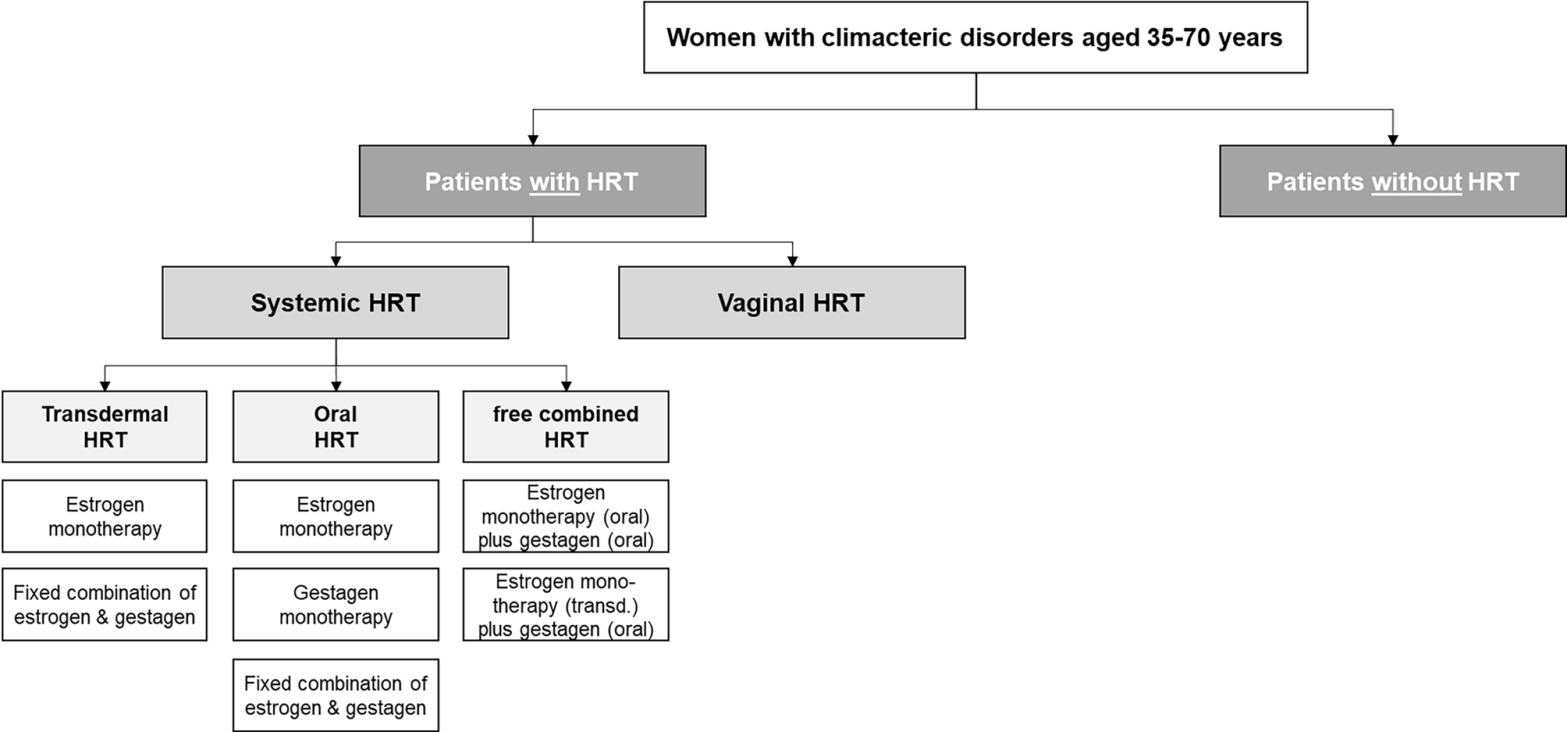
Classification of Cohort 1) with hormone replacement therapy (HRT) according to systemic (transdermal/oral/free combination1) and vaginal

The healthcare research is based on an anonymised, age- and gender-adjusted SHI claims data set of the Institute for Applied Health Research Berlin GmbH (InGef) with approximately four million insured persons per year and represents a representative sample of approximately six percent of the German population [27]. In a descriptive, retrospective longitudinal study of two consecutive years of observation, care patterns of female patients aged 35–70 with an initial diagnosis of climacteric disorder were mapped over a six-year period (2013–2018). The study population was divided into cohort 1) patients on HRT and cohort 2) patients not on HRT. Cohort 1) was further subdivided into subgroups based on the respective dosage form of the prescribed HRT. Only those prescriptions issued in the outpatient sector were considered. (Fig. 1). A two-group pre-post study design was used – for cohort 1) observation started after the first coded diagnosis and the follow-up observation started after the first prescription of HRT, whereas for cohort 2) patients were observed both before and after the initial coded diagnosis.

Patients aged 35–70 years were included in this healthcare study based on diagnosis coding according to the ICD-10-German Modification (GM) classification system (Table 1). Patients, who had a climacteric disorder as a confirmed outpatient diagnosis in at least two different quarters in the index year of 2014 (period between Q1 2014 and Q4 2018) or as an inpatient main or secondary diagnosis, were included in this study. It was stipulated that in the 365 days prior to the index period (2014), patients must not have had any of the predefined ICD-10-GM diagnoses documented or received an HRT prescription (baseline). Outpatient prescriptions were considered according to DIMDI ATC classification.

**Table 1:** Number and 95% confidence interval (CI) of patients with specific ICD-10-GM diagnosis at index and stratified per age group

ICD-10-GM Codes^1^		description	Age group	N at risk age group 35–70	N with diagnosis age group 35–70	N per 100.000 age group 35–70	95% UCI	95% LCI
N95 N93 N92 N91.1 N91.2 N91.4 N91.5	E34.9 E28.3 E28.8 E28.9 E89.4 Z90.7^2^	Inclusion criteria (total)	35–40	87,983	10,398	11,818	11,592	12.048
			41–45	90,270	11,192	12,398	12,170	12,630
			46–50	107,500	16,943	15,761	15,524	16,000
			51–60	178,121	27,126	15,229	15,048	15,411
			61–69	133,717	16,043	11,998	11,813	12,185
			70	15,513	1083	6981	6572	7410
			Total	613,104	82,785	13,503	13,411	13,595
N95		Climacteric disorder as primary diagnosis	35–40	87,983	313	356	317	397
			41–45	90,270	1547	1714	1629	1801
			46–50	107,500	7270	6763	6,608	6920
			51–60	178,121	22,378	12,563	12,399	12,729
			61–69	133,717	14,477	10,827	10,651	11,004
			70	15,513	984	6343	5953	6752
			Total	613,104	46,969	7661	7592	7730

^1^*N95* Climacteric disorder, *N93* Other abnormal uterine or vaginal bleeding, *N92* Menstruation that is too heavy, too frequent, or irregular, *N91.1* Secondary amenorrhea, *N91.2* Amenorrhea, unspecified, *N91.4* Secondary oligomenorrhea, *N91.5* Oligomenorrhea, unspecified, *E34.9* Endocrine disorder, unspecified, *E28.3* Primary ovarian failure, *E28.*8 Other ovarian dysfunction, *E28.9* Other ovarian dysfunction, *E89.4* Ovarian failure after medical measures, *Z90.7* Loss of one or more genital organs

^2^Factors that influence the state of health and lead to the use of the health system

## Results

### Patient characteristics (SHI claims data)

**Fig. 2:**
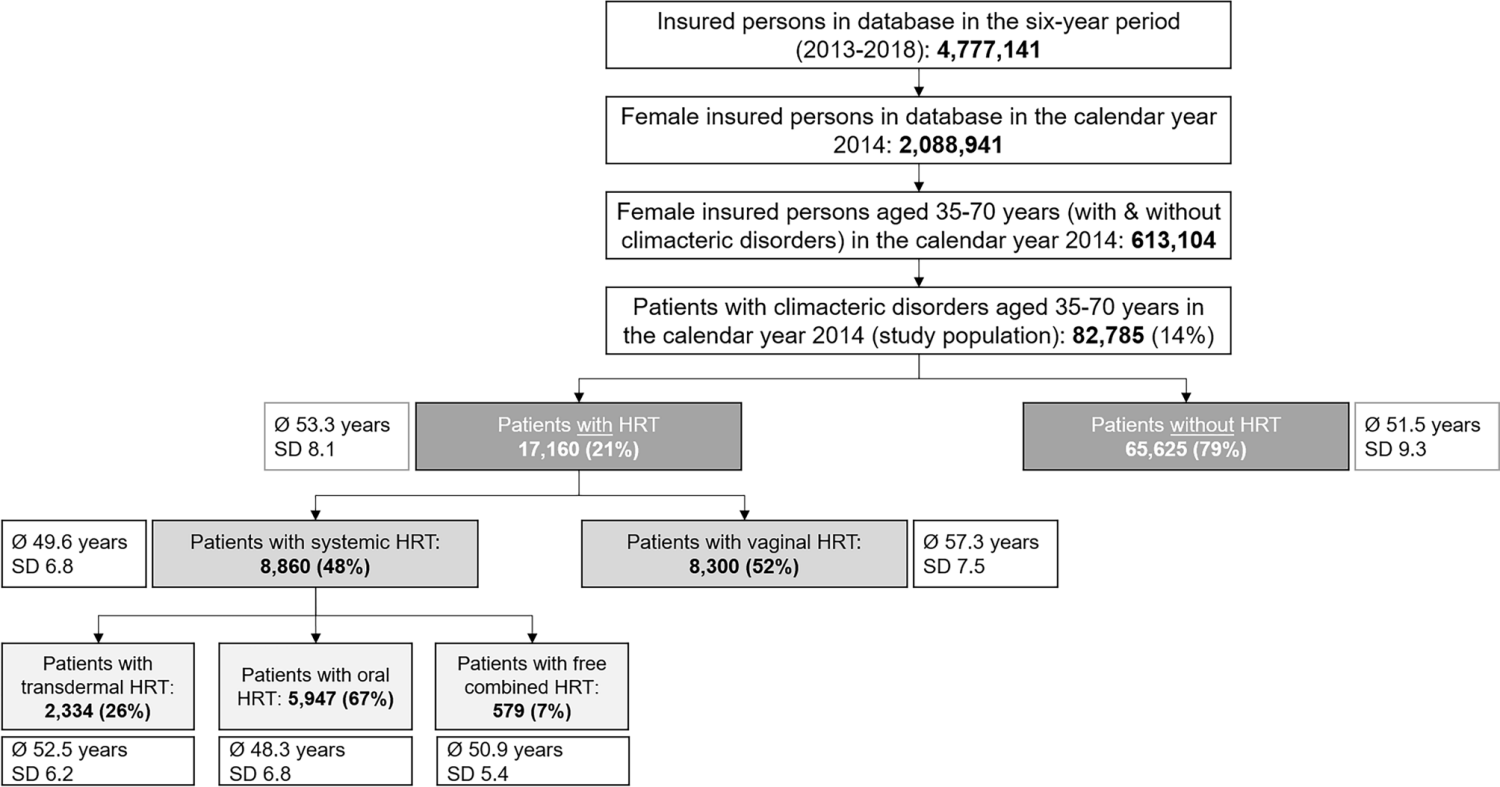
Sample description based on a patient flow diagram

Out of a total of 2,088,941 women with statutory health insurance, 613,104 women aged 35–70 years with and without climacteric disorder as an ICD-10-GM diagnosis were identified in the database in the calendar year 2014. Of these, 82,785 were patients (14%) with a confirmed initial diagnosis of climacteric disorder (Fig. 2). The administrative incidence in Germany was 4.0%; extrapolated, this corresponds to a total of 1.6 million women.

Patients in this study were identified predominantly by the N95 code (climacteric disorders) (*n* = 46,969). The highest incidence could be observed for the N95 code with 7,661 patients per 100,000 (95% confidence interval, 95% CI 7592–7730). The majority of women, who received N95 coding, were between 51–60 years of age (48%; *n* = 22,378) or older (61–69 years; 31%; *n* = 14,477) (Table 1). Based on the study population (*n* = 82,785), 65,625 patients (79%) were assigned to cohort 2) without HRT and 17,160 patients (21%) were assigned to cohort 1) with HRT. Thus, overall, the majority of patients did not receive HRT. In terms of the choice of HRT (systemic versus vaginal), the distribution was nearly 50:50. When systemic HRT was prescribed, it was primarily oral (67%), followed by the transdermal dosage form (26%) (Fig. 2).

### Disease burden

#### Menopausal women survey

In the forsa survey, two-thirds of women between the ages of 45 and 60 said they felt their quality of life was impaired by menopausal symptoms. One in seven women (14%) even felt severely restricted. More than a third of women living in Germany (37%) said that their health had become worse/much worse during perimenopause. Hot flashes (72%), sleep disturbances (51%), mood swings (38%), dry skin/mucous membranes (38%), and exhaustion (32%) were among the most common complaints. Despite this, 68% of women did not consider HRT until their symptoms became acute (Table 2).

**Table 2:** Number and share of patients with specific ICD-10-GM diagnosis as disease during follow-up within two years

ICD-10-GM Codes	Description	Baseline (365 days before diagnosis)						Follow-up (total)					
		Total population		Cohort with HRT		Cohort without HRT		Total population		Cohort with HRT		Cohort without HRT	
		*N* = 82,785		*N* = 17,160		*N* = 65,625		*N* = 82,619		*N* = 17,160		*N* = 65,459	
		*N*	%	*N*	%	*N*	%	*N*	%	*N*	%	*N*	%
A The comorbidities were assigned based on categories of menopause rating scale II (MRS II):													
Physical disabilities (somatoforme-vegetative disorders)													
M54	Back pain	32,731	39.5	7,741	45.1	24,990	38.1	44,697	54.1	10,313	60.1	34,384	52.5
N9511^1^	Conditions related to menopause and climacteric (inc. symptoms such as hot flashes, insomnia, headache, poor concentration associated with menopause)	0	0.0	0	0.0	0	0.0	18,043	21.8	5,230	30.5	12,813	19.6
G43	Migraine	7,432	9.0	1,840	10.7	5,592	8.5	9,278	11.2	2,288	13.3	6,990	10.7
M25.5	Joint pain	6,563	7.9	1,619	9.4	4,944	7.5	12,696	15.4	2,952	17.2	9,744	14.9
G47	sleep disorders	5,022	6.1	1,295	7.5	3,727	5.7	9,515	11.5	2,497	14.6	7,018	10.7
R51	Headache	4,480	5.4	1,098	6.4	3,382	5.2	7,700	9.3	1,905	11.1	5,795	8.9
R42	Dizziness	4,393	5.3	1,069	6.2	3,324	5.1	8,255	10.0	1,994	11.6	6,261	9.6
G44	Other headache syndromes	2,309	2.8	585	3.4	1,724	2.6	3,547	4.3	916	5.3	2,631	4.0
F51	Non-organic sleep disorders	890	1.1	232	1.4	658	1.0	2,188	2.6	578	3.4	1,610	2.5
R61	Hyperhidrosis	729	0.9	197	1.1	532	0.8	1,759	2.1	485	2.8	1,274	1.9
F45.40	Persistent somatoform pain disorder	716	0.9	212	1.2	504	0.8	1,452	1.8	383	2.2	1,069	1.6
H81.3	Other peripheral dizziness	302	0.4	75	0.4	227	0.3	488	0.6	109	0.6	379	0.6
H82	Vertigo Syndromes in Diseases Classified Elsewhere	39	0.05	13	0.1	26	0.04	73	0.1	19	0.1	54	0.1
Mental and behavioral disorders													
F43	Reactions to severe stress and adjustment disorders	8,025	9.7	1,862	10.9	6,163	9.4	14,422	17.5	3,409	19.9	11,013	16.8
F48.0	Neurasthenia	3,885	4.7	912	5.3	2,973	4.5	6,644	8.0	1,531	8.9	5,113	7.8
R53	Malaise and fatigue	3,571	4.3	796	4.6	2,775	4.2	7,249	8.8	1,674	9.8	5,575	8.5
Z73	Problems related to difficulty coping with life	1,820	2.2	403	2.3	1,417	2.2	3,458	4.2	871	5.1	2,587	4.0
R45	Symptoms affecting mood	1,518	1.8	369	2.2	1,149	1.8	2,976	3.6	759	4.4	2,217	3.4
G93.3	chronic fatigue syndrome	240	0.3	57	0.3	183	0.3	505	0.6	115	0.7	390	0.6
F38	Other mood disorders	48	0.1	13	0.1	35	0.1	109	0.1	27	0.2	82	0.1
F06.6	Organic, emotionally labile [asthenic] disorder	19	0.02	6	0.03	13	0.02	31	0.04	6	0.03	25	0.04
B. Diseases of the genitourinary system													
L85.3	Xeros Cutis (inkl, Xerodermie—trockene Haut)	350	0.4	84	0.5	266	0.4	848	1.0	192	1.1	656	1.0
F52.0	Lack or loss of sexual desire	173	0.2	39	0.2	134	0.2	674	0.8	238	1.4	436	0.7
F52.1	Sexual aversion and lack of sexual satisfaction	< 5	< 5	< 5	< 5	< 5	< 5	20	0.02	7	0.04	13	0.02

^1^N95.1 Coding was excluded in baseline

#### SHI claims data

Predefined concomitant diseases as well as climacteric complaints were investigated. Based on the total collective (*n* = 82,619), 49.3% of patients (*n* = 40,695) suffered from depressive disorders and/or behavioural disorders (F40-F45/F32-F33) and 36.2% (*n* = 29,867) from musculoskeletal disorders (M80-M81/M05-M19) during the two-year follow-up period. Regarding the studied complaints, which were based on the categories of the Menopause Rating Scale (MRS) II (1, 29), back pain (M54) with 54.1% (*n* = 44,697), conditions related to the menopause, such as hot flashes, insomnia, headache and lack of concentration (N95.1), with 21.8% (*n* = 18,043), reactions to severe stress and adjustment disorders (F43) with 17.5% (*n* = 14,422), joint pain (M25.5) with 15.4% (n = 12,690), sleep disorders (G47) with 11.5% (*n* = 9515) and migraine (G43) with 11.2% (*n* = 9278) were among the most common physical and psychological complaints. The proportion of patients with each concomitant condition was higher in cohort 1) with HRT. In this cohort, the proportion of patients with N95.1 coding decreased from 22.4% (*n* = 3838) in the first year of observation to 21.5% (*n* = 3692) in the second year of observation. The whole cohort shows 12.0% at baseline and 16.5% in the follow-up prescriptions of psychoanaleptics (incl. antidepressants etc.), with 15.3 and 21.7%, respectively, in the cohort with HRT and 11.1 and 15.2%, respectively, in the cohort without HRT. During the follow-up period, 45.5% of the whole cohort had a psychological examination, which comprised 58.0% of the cohort with HRT and 42.3% of the cohort without HRT.

### Change of physician

#### Menopausal women survey

More than one-third of the women (37%) felt that their gynaecologist provided mediocre or poor/very poor advice on menopause. With regard to therapies, 50% felt moderately or poorly/very poorly informed.

#### SHI claims data

**Fig. 3:**
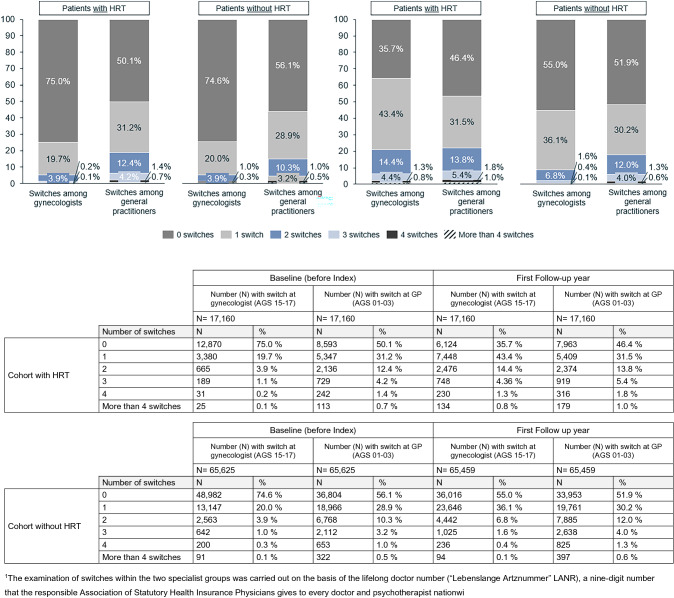
Proportion of patients and number of physician changes* (within the specialty group of gynaecologists and family physicians) at baseline (left) and in the first year of follow-up (right)

The majority of patients with and without HRT (75% in each case) did not change their general practitioner or gynaecologist before a diagnosis of the climacteric disorder (baseline). Based on the cohort with HRT, the proportion of patients, who changed their gynaecologist at least once or twice, increased in the first year of follow-up (1 change = 43%; 2 changes = 14%; ≥ 3 changes = 6%). This is significantly higher than the observed switching frequency in the cohort without HRT (1 switch = 36%; 2 switches = 7%; ≥ 3 switches = 2%) (Fig. 3). Family physicians, on the other hand, were changed less in both the cohort with HRT and the cohort without HRT over the observation period. HRT was prescribed on average 1½ years after diagnosis. The use of systemic HRT (MW 15.9; SD 14.9 months) was started earlier compared with vaginal HRT (MW 19.5; SD 16.2 months). Patients receiving systemic HRT (MW 49.6; SD 6.8 years) were significantly younger than women receiving vaginal HRT (MW 57.3; SD 7.5 years) (Fig. 2).

### Incapacity for work (AU)

#### SHI claims data

Climacteric disorders were not considered an obligatory incapacity for work (AU) diagnosis. The proportion of employed women with at least one day of certified incapacity for work during the two-year follow-up period was 36.3% in women without ICD-10 coding of climacteric disorder, while the proportion of patients with the coded climacteric disorder was 41.0%. The average number of days of sick leave in the first year of observation in women without coded climacteric disorder was 26.5 (SD 42.0), and is thus comparable to the 28.0 days (SD 43.1) in patients with coded climacteric disorder. With respect to sickness benefits (AU for > 6 weeks), the overall proportion of patients with the coded climacteric disorder was 6.1% during the 2-year follow-up period, which was higher than the 4.9% for the group of women without the coded climacteric disorder. A top-5 ranking of AU diagnoses showed that back pain (M54) was among the most common conditions to be substantiated. In the first year of observation, the proportion was 3.7% in the female group without coded climacteric disorder and 5.0% in female patients overall.

### Therapies and costs

#### SHI claims data

Overall, agents from the group of antiphlogistics and antirheumatics (M01) were prescribed in 47.5% of the total collective of patients with the coded climacteric disorder (*n* = 39,280), followed by analgesics (N02) with 31.4% (*n* = 25,978) and psychoanaleptics (N06 incl. SRRIs and SNRIs) with 16.5% (*n* = 13,670). In addition, 45.5% (*n* = 37,614) of patients were receiving outpatient psychotherapeutic treatment during the two-year follow-up period.

Total costs per patient were slightly lower for the cohort without HRT (observation year 1: 2,220.01 euros p.a.; observation year 2: 2300.65 euros p.a.) compared to the cohort with HRT (observation year 1: 2497.56 euros p.a.; observation year 2: 2501.07 euros p.a.). However, higher medication costs per patient were recorded in the cohort without HRT (observation year 1: 551.21 euros p.a.; observation year 2: 586.91 euros p.a.) compared to the cohort with HRT (observation year 1: 531.04 euros p.a.; observation year 2: 566.20 euros p.a.).

## Discussion

The linking of primary data (survey) with SHI claims data (secondary data) used in this study allows a comprehensive insight into the care of menopausal women since the patient perspective is included in the holistic view in addition to the documented diagnoses and services provided. The use of SHI claims data is associated with specific limitations since the informative value depends not only on the differentiability of the underlying coding system, but also on the coding quality in everyday clinical practice. For this reason, in addition to the specific N95 code (climacteric disorders), other ICD-10-GM diagnoses were considered to help identify menopausal women, to avoid overlooking any patients, if possible. In the quantitative study, the majority of the patients aged 51–69 years were identified by N95 coding. The study conducted by the Central Institute for Statutory Health Care in Germany confirmed that in 45.3% of cases menopausal symptoms were most frequently billed via N95 in gynaecology practices [28]. Nevertheless, the N92 code ("Menstruation too heavy, too frequent or irregular") also plays an important role in identifying younger patients (26%; *n* = 21,889), although it is not possible to determine from the coding alone whether the diagnosis is a true perimenopausal diagnosis or whether the cases coded with N92 are related to other diseases (e.g. hormonal disorders, oncological diseases).

Findings from the epidemiological data collection show that out of more than half a million women aged 35–70 years, and with statutory health insurance, (*n* = 613,104), 14% (*n* = 82,785) had menopausal disorders documented as a first diagnosis in 2014. Considering that, according to the forsa survey, two-thirds of women aged 45–60 feel that their quality of life is impaired by menopausal symptoms, a high discrepancy can be observed, which would suggest undercoding or miscoding.

Although there are methodological flaws in the WHI study [6–8], there has been a marked uncertainty and change in prescribing behavior among practitioners that continues to persist [20]. It is therefore not surprising that, according to our evaluation, only 21% of patients receive HRT despite their menopausal symptoms and that they also have to "wait" about 18 months for it. Even though the proportion with HRT increases in the patient group with exclusive N95 coding (37%), the data suggest an existing underuse. This proportion is comparable to the Robert Koch Institute DEGS1 study, in which 35.5% of women reported using HRT [28]. It should be noted that SHI claims data only capture services that were billed through SHI; completeness of data is thus limited since there will be medical services provided that are not billed through SHI (e.g. OTC preparations, hormone magistral prescriptions) [29]. In addition, combination therapies of systemic and vaginal HRT were not investigated in this study.

Based on the SHI claims data and the forsa survey, the female patient population may experience a higher burden of disease, if symptoms remain untreated. In addition to mental disorders, musculoskeletal diseases also play an important role. The high level of suffering is evident from the concomitant illnesses that are closely related to the use of psychotherapy. In addition, 21.8% of patients suffered from conditions related to menopause and climacteric (N95.1). The high prevalence of comorbid psychological disorders and other pain syndromes suggests the need for comprehensive treatment options, especially since it is known that in patients with menopausal/vasomotor symptoms (VMS), the benefits of HRT generally outweigh the comparatively small risks associated with it [7, 9]. Nevertheless, according to the survey, most women (68%) do not consider the possibility of HRT until symptoms become acute. In addition, 50% of the women surveyed felt only moderately to poorly/very poorly informed about treatment options. The increased need for information could also be due to the ongoing discussion about the (breast) cancer risk under HRT. The suffering is reinforced by the prescription of specific concomitant medications (analgesics and psychoanaleptics). In particular, higher medication costs per patient (p.a.) were recorded in the cohort without HRT. Also, for example, in the American study by Sarrel et al. [23], women with untreated VMS showed not only significantly higher direct costs per patient (MW 1,346 US dollars p.a.) and higher indirect costs (57% loss of productivity) but also a significantly higher utilisation of healthcare resources (82% higher for all physician visits; 121% for VMS-related physician visits) than women in the control cohort.

In conclusion, it can be assumed that the long period without therapy and the increased number of changes in physicians are due, among other things, to the general uncertainty on the part of patients and physicians, existing gaps in information about therapy options, the patient’s wish to obtain a second medical opinion, the woman's inner psychological conflicts, the initial use of herbal preparations and the increase in complaints in the period after diagnosis. Findings from healthcare research point to the need for increasing awareness and providing early and informative education on HRT. Especially since, according to current knowledge, untreated menopausal symptoms are associated with higher healthcare costs [17, 23, 24], a decrease in work productivity [17, 23, 25, 26, 30] and increased physician visits [23, 25, 26]).
